# Distribution and Potential Indicators of Hospitalized Cases of Neurocysticercosis and Epilepsy in Ecuador from 1996 to 2008

**DOI:** 10.1371/journal.pntd.0004236

**Published:** 2015-11-18

**Authors:** Lenin Ron-Garrido, Marco Coral-Almeida, Sarah Gabriël, Washington Benitez-Ortiz, Claude Saegerman, Pierre Dorny, Dirk Berkvens, Emmanuel Nji Abatih

**Affiliations:** 1 Universidad Central del Ecuador, Centro Internacional de Zoonosis (CIZ), Ciudadela Universitaria, Quito, Ecuador; 2 Department of Biomedical Sciences, Institute of Tropical Medicine, Antwerp, Belgium; 3 Research Unit of Epidemiology and Risk Analysis applied to the Veterinary Sciences (UREAR-ULg), Fundamental and Applied Research for Animal and Health (FARAH), Faculty of Veterinary Medicine, University of Liège, Liège, Belgium; 4 Universidad de las Américas, Escuela de Medicina Veterinaria y Zootecnia, Quito Ecuador; 5 Ghent University, Faculty of Veterinary Medicine, Merelbeke, Belgium; Universidad Nacional Autónoma de México, MEXICO

## Abstract

**Background:**

Epilepsy is one of the most common signs of Neurocysticercosis (NCC). In this study, spatial and temporal variations in the incidence of hospitalized cases (IHC) of epilepsy and NCC in Ecuadorian municipalities were analyzed. Additionally, potential socio-economic and landscape indicators were evaluated in order to understand in part the macro-epidemiology of the *Taenia solium* taeniasis/cysticercosis complex.

**Methodology:**

Data on the number of hospitalized epilepsy and NCC cases by municipality of residence were obtained from morbidity-hospital systems in Ecuador. SatScan software was used to determine whether variations in the IHC of epilepsy and NCC in space and time. In addition, several socio-economic and landscape variables at municipality level were used to study factors intervening in the macro-epidemiology of these diseases. Negative Binomial regression models through stepwise selection and Bayesian Model Averaging (BMA) were used to explain the variations in the IHC of epilepsy and NCC.

**Principal findings:**

Different clusters were identified through space and time. Traditional endemic zones for NCC and epilepsy, recognized in other studies were confirmed in our study. However, for both disorders more recent clusters were identified. Among municipalities, an increasing tendency for IHC of epilepsy, and a decreasing tendency for the IHC of NCC were observed over time. In contrast, within municipalities a positive linear relationship between both disorders was found. An increase in the implementation of systems for eliminating excrements would help to reduce the IHC of epilepsy by 1.00% (IC_95%_; 0.2%–1.8%) and by 5.12% (IC_95%_; 3.63%-6.59%) for the IHC of NCC. The presence of pig production was related to IHC of NCC.

**Conclusion/Significance:**

Both disorders were related to the lack of an efficient system for eliminating excrements. Given the appearance of recent epilepsy clusters, these locations should be studied in depth to discriminate epilepsies due to NCC from epilepsies due to other causes. Field studies are needed to evaluate the true prevalence of cysticercosis in humans and pigs in different zones of the country in order to better implement and manage prevention and/or control campaigns.

## Author Summary

*T*. *solium* neurocysticercosis is considered the most important parasitic disease of the central nervous system in humans; it is estimated to be responsible for at least one third of acquired epilepsies in developing countries. In Ecuador, the relationship between acquired epilepsy and neurocysticercosis remains unclear due to different factors such as, the lack of specialized health care personnel, appropriate diagnostic techniques and the fact that acquired epilepsy is characteristic of many other infectious and non-infectious diseases in the endemic zones of the country. In this study, spatio-temporal information and potential socio-economic indicators were studied for the number of hospitalized neurocysticercosis and epileptic cases in the country in order to locate and characterize important clusters in space and time. This study identified traditional endemic clusters in the highlands for both conditions as well as new clusters appearing in recent years in other zones not considered endemic. Also the incidence of hospitalized cases of epilepsy and neurocysticercosis were significantly higher in urban zones, probably due to a better access to health facilities. The presence of systems for excrement disposal was significantly associated with a reduction in the incident cases for both epilepsy and neurocysticercosis. More studies are needed to evaluate the true prevalence of neurocysticercosis associated epilepsy in humans and cysticercosis in pigs around the country in order to better implement and manage control campaigns.

## Introduction

Humans are the definitive hosts of *Taenia solium* harboring the intestinal adult tapeworm, which causes taeniasis [1;2]. Humans acquire the tapeworm through consumption of improperly cooked infected pork. The intermediate pig host gets infected by ingestion of parasite eggs, passed in the stool of a tapeworm carrier. The metacestode larval stage establishes in the pig’s muscles, brain and other tissues (cysticercosis) [2]. Unfortunately, humans can also serve as dead-end intermediate hosts by accidentally ingesting parasite eggs and developing the metacestode larval stage [2]. In humans, the parasite tends to locate in the central nervous system (neurocysticercosis (NCC)) causing a variety of neurological symptoms, such as seizures, headache and in many cases epilepsy [3–5].

In developing countries, NCC is often an underrecognized and neglected disease [6;7]. The large variety of clinical signs and symptoms, and the inaccessibility of highly sensitive tests, like Computerized Tomography (CT) or Magnetic Resonance Imaging (MRI) (due to high costs involved and the unavailability of neuroimaging facilities), have contributed to the underreporting of NCC.

Different measures such as education, improvement of household sanitation, changes in meat inspection practices, identification and treatment of tapeworm carriers, mass drug administration and modifications in pig-rearing methods have proven, at least at short term, to be effective in lowering levels of transmission of NCC [8–10]. However, practices like free roaming pig management, clandestine marketing of living pigs and pork, open defecation, and the use of residual waters in irrigation [10;11], make the disease still prevalent in many regions.

In developing countries, *T*. *solium* NCC has been found to be the leading cause of acquired epilepsy. In fact, NCC was found to be responsible for at least half or a third of acquired epilepsy cases [12;13]. In Ecuador, an average of 480 and 1670 patients are hospitalized each year because of NCC and epilepsy, respectively [14]. Notification of hospitalized cases for both disorders is mandatory in Ecuador. Tools for diagnosing causes of acquired epilepsy, including parasitic and infectious diseases are available in the country; however, these diagnostic tools are not regularly used because of their high costs, thus, as in other NCC endemic countries, in Ecuador more than 82% of epilepsies do not have definitive diagnosis and the cause remains unknown [14–17].

Symptomatic and asymptomatic cases of NCC have been studied in urban [18–20] and rural [12;21–25] areas of Ecuador in different epidemiological studies. Endemic areas were found mostly in the highlands, where the population has a high exposure to the parasite as measured by specific antibody detection. Up to one third of the population is seropositive in some of these areas [22]. In some endemic communities in these highland areas, human cysticercosis active infections, as measured by antigen detection are present in up to five percent of the population [23]. However, in the past three decades, a decreasing trend in the incidence of hospitalized cases (IHC) of NCC has been observed in the country, apparently due to an improvement in sanitary conditions and the use of better diagnostic tools [20].

**Fig 1:**
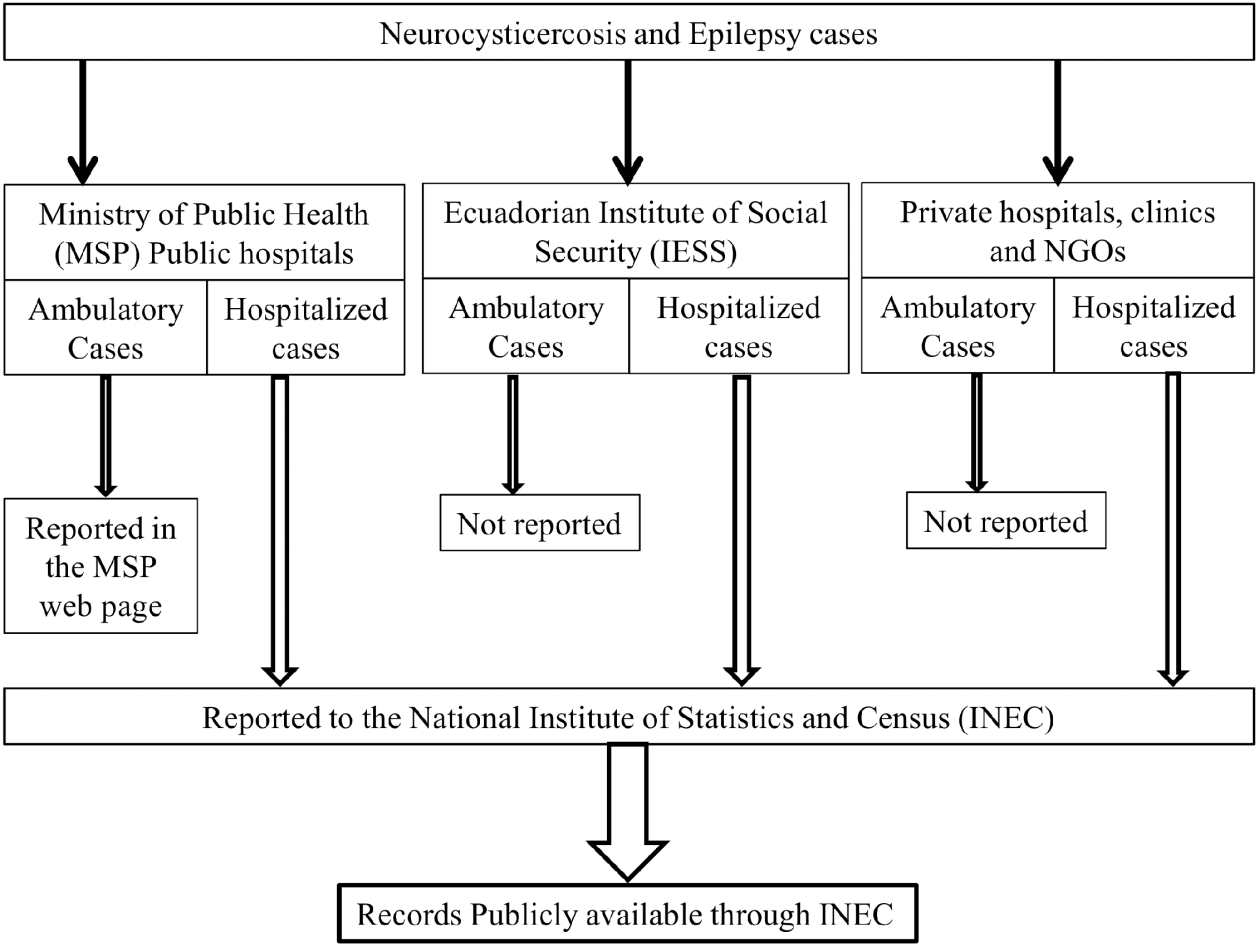
Neurocysticercosis and epilepsy reporting flow chart in the Ecuadorian health system. Legend: NGO: Non-governmental organization.

Epidemiological surveillance data in the Ecuadorian health status reports only include ambulatory cases from the public health care system. The public registers from the Ministry of Public Health are the only official health reports available. The consultations at any level attended in the public health system have been estimated to be around 30% of all the medical consultations in the country [26]. However, for the ambulatory cases the reporting is not always done properly, mainly due to the lack of synchronization among the different types of health care services in Ecuador [27]. Fig 1 explains the reporting system for NCC and epilepsy cases in Ecuador. Additionally, and in a parallel way, the National office of Statistics (INEC) collects all information on morbidity registered in hospitals and clinical centres (from public, private or social security systems) where patients are attended at the secondary health care level and also in case of emergencies irrespective of their underlying sources. In these registers, both NCC and epilepsy are frequent causes of hospitalization. Thus, epidemiological data generated by the Ecuadorian office of statistics offers the possibility of studying important variables related to the macro-epidemiology of NCC and epilepsy in Ecuador. Here, we use macro-epidemiology in terms of the determinants of disease, including economic, social, and climatological factors into national patterns in risk assessment [28].

In this study we aimed at identifying areas (municipalities) with a high IHC of NCC and epilepsy between 1996 and 2008. In addition, given the fact that the IHC of NCC and epilepsy have been related to several socio-economic, and landscape variables, we evaluated the macro-epidemiology of epilepsy and NCC in Ecuador at the municipality level. Finally, spatio-temporal analysis was implemented in order to investigate the distribution of the IHC of epilepsy and NCC in Ecuador.

## Materials and Methods

### Ethics statement

Ethical approval was not required for this study. All information used in this study came from public sources freely available on the referenced websites. Reports of human cases belonged to the public health surveillance system, the anonymity of clinical histories is guaranteed by legal mandate.

### Study region and data

The study unit was the municipality. The number of patients hospitalized in different institutions with diagnosis of epilepsy and NCC, from 1996 to 2008 was obtained from hospital morbidity and mortality databases managed by the National Office of Statistics (INEC)[14]. This time period was chosen because of the availability of digitized morbidity data (www.inec.gob.ec), and because data on agricultural and life conditions of the population were available for that time period [14;29]. Additionally, we did not consider the data of the period after 2008 because biases in the number of cases were expected given the fact that public health systems increased their coverage and became more accessible and free of charge, including the distribution of parasitic drugs [30]. The cases were identified through the ICD-10 codes for disease classification [31]. In Ecuador, the protocol to declare a patient with NCC is defined by the neurology department of hospitals. Briefly, the diagnosis of NCC follows the directions of the Del Brutto (2012) criteria. The diagnosis is based on patient’ clinical symptoms and signs (seizures, headache, dementia, hydrocephalous, among other neurological disorders), serology (detection of antibodies directed to *T*. *solium* metacestodes and/or circulating antigens of *T*. *solium* metacestodes in serum or cerebrospinal fluid) and imaging (CT, MRI) [32]. In the public sector there exist 39 neuroimaging facilities (CT-Scans) the majority of them are in the provincial capital cities (24 provinces). The private sector also has neuroimaging capacity but the number of scanners is unknown. The database has information about each hospitalized patient with cause-specific morbidity, hospital of attendance, and the place (parish or municipality) where the patient lives (access to official databases are included in S2).

All municipalities in the continental part of the country were included in this study (217 municipalities). The registers of the Galapagos Islands were excluded because they might distort the spatial analysis, although both disorders have also been reported there. Each record was designed to contain the total number of NCC and epilepsy cases, the total population, the year and the geographical coordinates of the centroid of each municipality. Time trends of hospitalized reported cases for both disorders were tested using Negative Binomial regression models. Furthermore, the relationship between the number of hospitalized cases of epilepsy and NCC was evaluated using correlation analysis on Log transformed data.

Additionally, data on several explanatory variables were gathered using governmental databases about several socio-economic indices, information from the agricultural census conducted in 2000, and climatological information from the National Hydrometeorological Service [33]. The information on explanatory variables possibly associated with NCC and epilepsy at municipality level were grouped into different classes. Climatic variables: (tropical or highlands) (ZONE), rainfall (RAIN) and number of days without precipitations (DRYD). For each municipality, values of RAIN and DRYD were inferred on the basis of 205 weather stations, and ordinary Kriging was used to interpolate values. The similarity with rainfall maps published by the National Hydrometeorological Service (INAMHI) let us choose the proper model [33]. Population variables: population number (POPULATION), percentage of indigenous population (%INDG), and percentage of rural population (%RURAL); Educational level: mean number of years of schooling (SCHOOL), mean number of years of schooling for farmers in the municipality (FSCHOOL), and percentage of farms receiving technical assistance (TECHASSIS); Sanitary conditions: % of families with piped water (TUBWAT), % of dwellings with systems for eliminating excrements (EXCR), and physicians per 10,000 inhabitants (PHYS); Poverty indices such as: percentage of families with unsatisfied basic necessities (UBN) (Number of people or families that live under poverty conditions with respect to the population in a specific year, referring to the lack of dwelling, health, education and employment [29]), and percentage of people under extreme poverty (EXTPOOV); Livestock: percentage of agriculture land dedicated to pastures (%GLS) and pig population (PIG)[29]. In Ecuador, the majority (58.8%) of the pig population is raised under the traditional husbandry system if we consider smallholder producers in Ecuador as those farmers owning ≤10 pigs [34]. The agricultural office of geographical information systems provided the map showing the political division of the country at municipality level. A description of all the variables considered for this study is presented in Table 1(access to official databases are included in S2).

**Table 1:** Socio-economic and demographic factors used to model the incidence risk of hospitalized human cases of epilepsy and neurocysticercosis in Ecuador from 1996 to 2008.

Variable	Description
POPULATION	Population
EPI	Number of epilepsy cases (1996–2008)
CYS	Number of NCC cases (1996–2008)
Zone	Tropical (1) or temperate zone(0)
RAIN	Rainfall during the year
DRYD	Number of days during a year with precipitations ≤ 1mm
%GLS	Percentage of agriculture land dedicate to pastures
PIG	Number of pigs (Criollo breed)
SCHOOL	Average number of years on formal education in people > = 24 years old
PHYS	Number of physicians per 10,000 inhabitants
TUBWAT	Percentage of dwellings with piped water
EXCR	Percentage of dwellings with some kind of system for eliminating excrements.
UBN	Percentage of families with unsatisfied basic necessities
EXTPOOV	Percentage of extreme poverty
%INDG	Percentage of indigenous population
%RURAL	Percentage of rural population.
TECHASSIS	Percentage of land with technical assistance
FSCHOOL	Years of education of farmers> 24 years
*N*	217 municipalities

### Space-time analysis

Space-time analysis was used to determine whether municipalities with high incidence of hospitalized cases (IHC) of epilepsy and NCC are clustered in space and in time [35;36]. The space-time scan software [35] was used to search, and test for significance and identify approximate locations of areas with an increased risk for the occurrence of NCC and epilepsy cases. The analysis was run in SatScan Software v9.4, with case file as the number of hospitalized reported cases, population file as the estimated total number of individuals in each municipality per year and as the coordinate file, the latitudes and longitudes of the centroids of each municipality. The spatial dimension varied from 0 up to 25% of the total number of centroids in the study region. The temporal dimension was established with a maximum of up to 50% of the study period with a time precision of 1 year. Poisson-distribution was used to contrast the number of cases in the scanning of areas. Space-time clustering was assessed by comparing the iRR (incidence rate ratio) of epilepsy and NCC IHC within a specific area and time in contrast to an expected iRR of hospitalized NCC and epilepsy cases if their incidences were randomly distributed. The cylinder with the maximum likelihood ratio was selected as the most likely cluster (Primary cluster), and others no overlapping significant clusters were also selected. The significance of identified space-time clusters was tested using the likelihood ratio test statistic and p-values of the test were obtained through Monte Carlo simulations (999). The significance was arbitrated at the 5% level. All significant clusters were visualized using QGIS software (version 2.8).

### Regression analysis

Multivariable-count regression models were used to assess the relative contribution of different socio-economic and demographic variables to the IHC of epilepsy and NCC from 1996 to 2008 across the country. For the case of hospitalized NCC cases, an excess of zero cases were observed. For this reason, Zero inflated negative binomial models (ZINB) were used to explain the over-abundance of zero cases [37].

A manual forward stepwise procedure was implemented to select the set of independent variables that describe the number of NCC hospitalized cases or the probability of not observing any hospitalized case of NCC using the *zinb* command in STATA, version 12 software (StataCorp LP, College Station, Texas). The procedure started with the null model (with no covariates), and subsequently, covariates were added and evaluated for their importance. The Akaike Information Criteria (AIC) was used as the calibrating parameter and models with lower AIC values and few parameters were preferred. Two models were considered to be significantly different whenever the difference in AIC was greater than 3 [38]. In the forward selection procedure, each covariate was added in either the linear predictor for the count part or in the *logit* function for the absence of the disease, and the covariate with the best explanation preserved for the second round. If the addition of a covariate improved the model explanation through the reduction in AIC, the variable was captured and the process was repeated until the AIC value could not be reduced further [39]. The significance level for the covariates in the model was set at 0.05. For the case of NCC Vuong’s test was applied in order to evaluate if zero-inflation was more appropriate as compared to the standard negative binomial model.

To assess the influence of the selected indicators on the IHC of epilepsy, a Poisson regression model was used, and due to the presence of over-dispersion, Negative Binomial models were also evaluated through a likelihood ratio test for over-dispersion using the function *odTest* (in *pscl* package, in the R software) to test the null hypothesis that the restriction implicit in the Poisson model is true [40]. The approach of bidirectional elimination of variables was applied for variable selection, which also used the AIC as calibrating parameter. The functions *glm*.*nb* and *stepAIC*, in the *MASS* package under the R environment were used [41].

In addition, for the study of epilepsy, Bayesian model averaging (BMA) was used in order to deal with the uncertainty about the “correct” model [42]. BMA chooses the better model according to the best posterior probabilities among the models using the Occam’s window principle [38]. The inference about the explanatory variables in the best model is expressed as posterior effect probabilities, which indicate evidence of the importance of the effects of each variable in the model. The function *bic*.*glm()* in the *BMA* package of the R software was used [43] with the specification that counts follow a quasi-Poisson distribution, and that variance increases with the square of the mean: an equivalent version of NB regression [44]. No explicit prior distributions for models and model parameters were assumed implying that all models were equally likely.

## Results

### Space-time analysis

**Fig 2:**
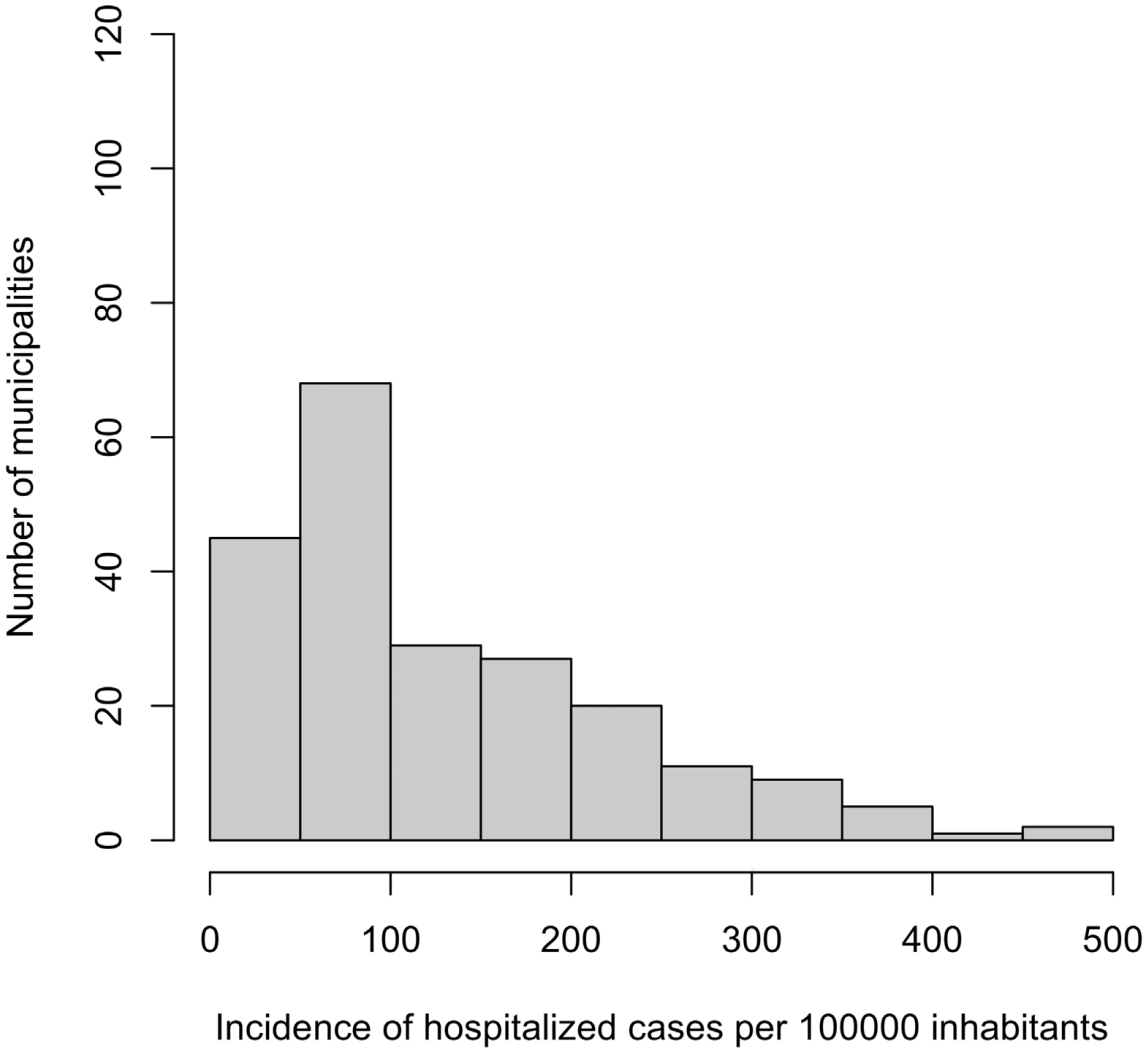
Histogram of the incidence of hospitalized epilepsy cases per 100,000 inhabitants between 1996 and 2008 in Ecuadorian municipalities.

**Fig 3:**
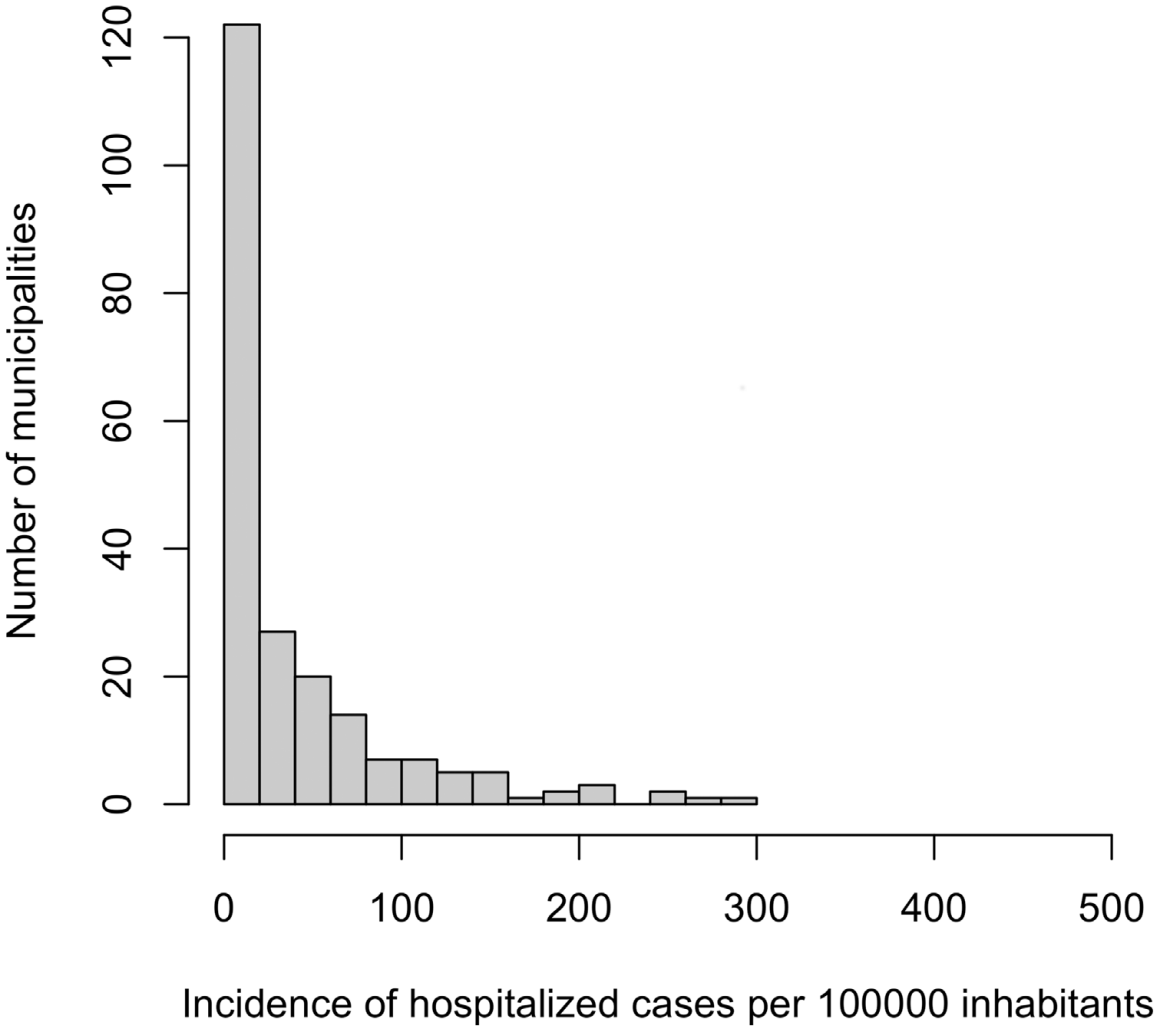
Histogram of the incidence of hospitalized neurocysticercosis cases per 100,000 inhabitants between 1996 and 2008 in Ecuadorian municipalities.

**Fig 4:**
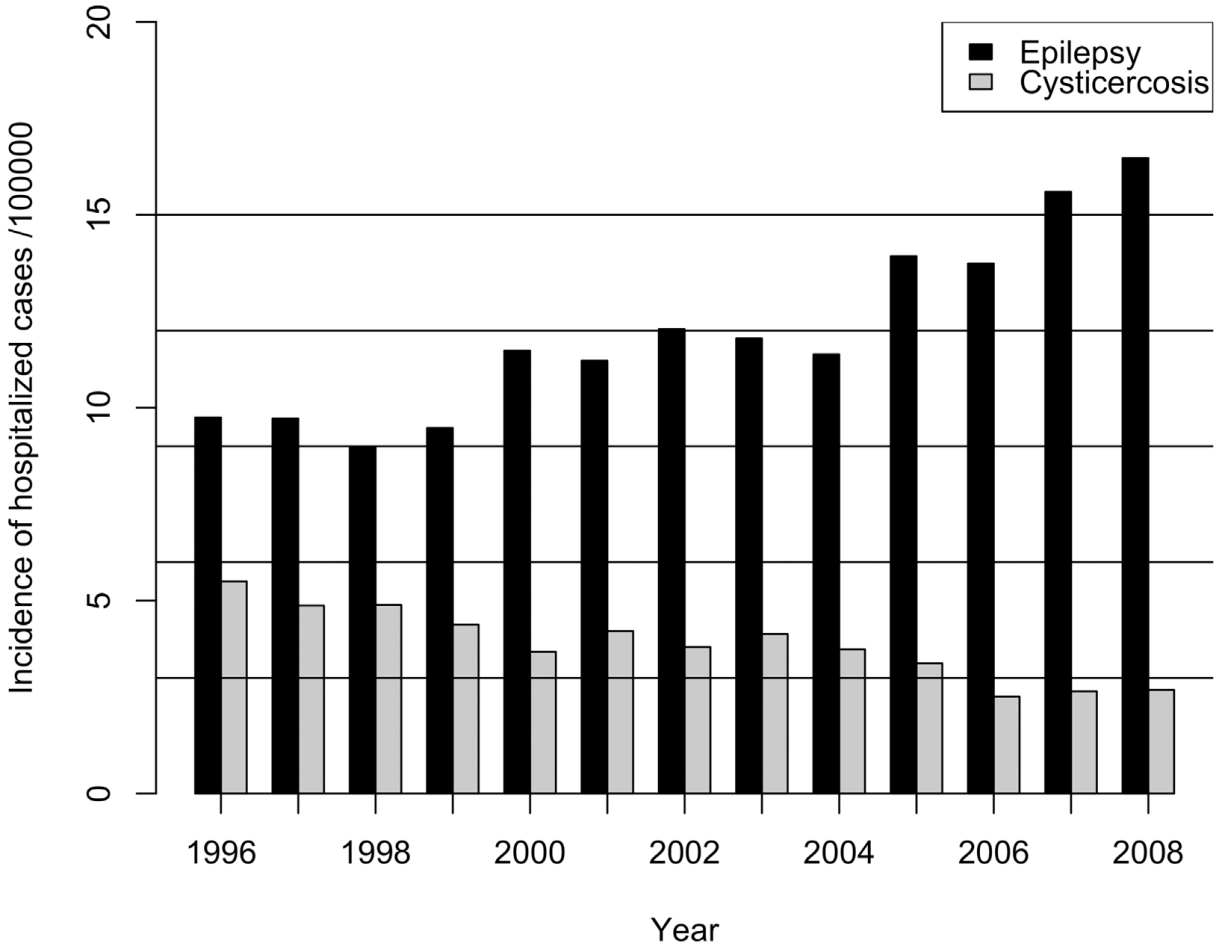
Incidence of hospitalized neurocysticercosis and epilepsy cases per 100,000 inhabitants in Ecuador between 1996 and 2008.

Figs 2 and 3 display the histogram of the cumulative incidence of hospitalized epilepsy (Fig 2) and NCC (Fig 3) cases, during the study period (between 1996 and 2008) over all the municipalities. For epilepsy, the distribution is more uniform compared to that of NCC; however, for NCC there are high proportions of zero cases throughout the municipalities. Fig 4 presents the Incidence of hospitalized cases (IHC) with both health problems over time in Ecuador. In the case of NCC, from 1996 to 2008, there was an overall decreasing trend with around 5 cases per 100,000 inhabitants in 1996 to around 3 cases per 100,000 inhabitants in 2008. The decreasing trend was statistically significant (p<0.001); thus, annually a reduction of 5.68% (IC_95%_: 4.5%-6.4%) in hospital cases is expected. In contrast, for the case of epilepsy, the incidence appeared to be slowly and steadily increasing from 1996 to 2008. The increasing trend was statistically significant (p<0.001). Annually an increase of 4.7% (%(IC_95%_: 3.7%-5.7%) in reported cases is expected. It was also observed that as long as the incidence of epilepsy increased through time, the annual IHC of NCC appeared to decrease slowly; and the IHC for epilepsy was always higher than that of NCC.

Table 2 presents a list of the top 15 municipalities with the highest IHC of epilepsy and NCC. It can be observed that municipalities with a high IHC of epilepsy did not necessarily have a high IHC of NCC. However, an overall (all years combined for each municipality), highly significant positive linear correlation (r = 0.78, IC_95%_(0.72–1.00), p<0.0001) was found on log(x+1) transformed number of hospitalized cases of epilepsy and NCC reported in each municipality. Similar significant positive linear trends within municipalities were observed for each year evaluated separately. The IHC of epilepsy varied from 0 to 505 cases per 100,000 with an average number of 127.1 cases. On the other hand, for NCC, the IHC varied from 0 to 282.54, and the average number of cases was 35.1. Out of the top 15 municipalities with high IHC for epilepsy, six also featured amongst the top 15 for municipalities with high IHC for NCC. It should be highlighted that Quito (capital city) presented the highest number of patients (1829 and 3123) in hospitals for NCC and epilepsy during the study period, but the rates were diluted because of the city’s large population size.

**Table 2:** Municipalities having the highest incidence of hospitalized cases (/100,000 inhabitants) of neurocysticercosis and of epilepsy from 1996 to 2008.

Municipality	Highest IHC for NCC		Municipality	Highest IHC for epilepsy	
	IHC NCC	IHC epilepsy		IHC NCC	IHC Epilepsy
Chinchipe	282.5	494.4	Paulo VI	0.0	505.0
Loja	241.6	327.4	Chinchipe	*	*
Quilanga	218.2	43.6	Zamora	*	*
Espíndola	215.8	273.0	Sta. Clara	0.0	396.2
El Tambo	206.0	218.2	El Chaco	130.4	391.3
Azogues	204.9	359.0	Sucúa	13.8	381.6
Zamora	183.6	472.7	Santiago	0.0	375.9
Cañar	171.7	268.1	Baños	0.0	367.1
Paltas	165.9	206.4	Azogues	*	*
Calvas	159.4	282.6	Sta. Rosa	28.1	341.1
Biblian	144.7	328.1	Biblian	*	*
Gonzanama	140.1	126.8	Loja	*	*
Cuenca	138.8	288.8	Morona	51.0	321.8
Riobamba	135.5	320.7	Riobamba	*	*
Ibarra	134.4	204.2	Penipe	61.0	308.0

Legend: NCC: Neurocysticercosis; IHC: Incidence of hospitalized cases

*: Data reported in the previous column

### Significant space-time clusters

**Fig 5:**
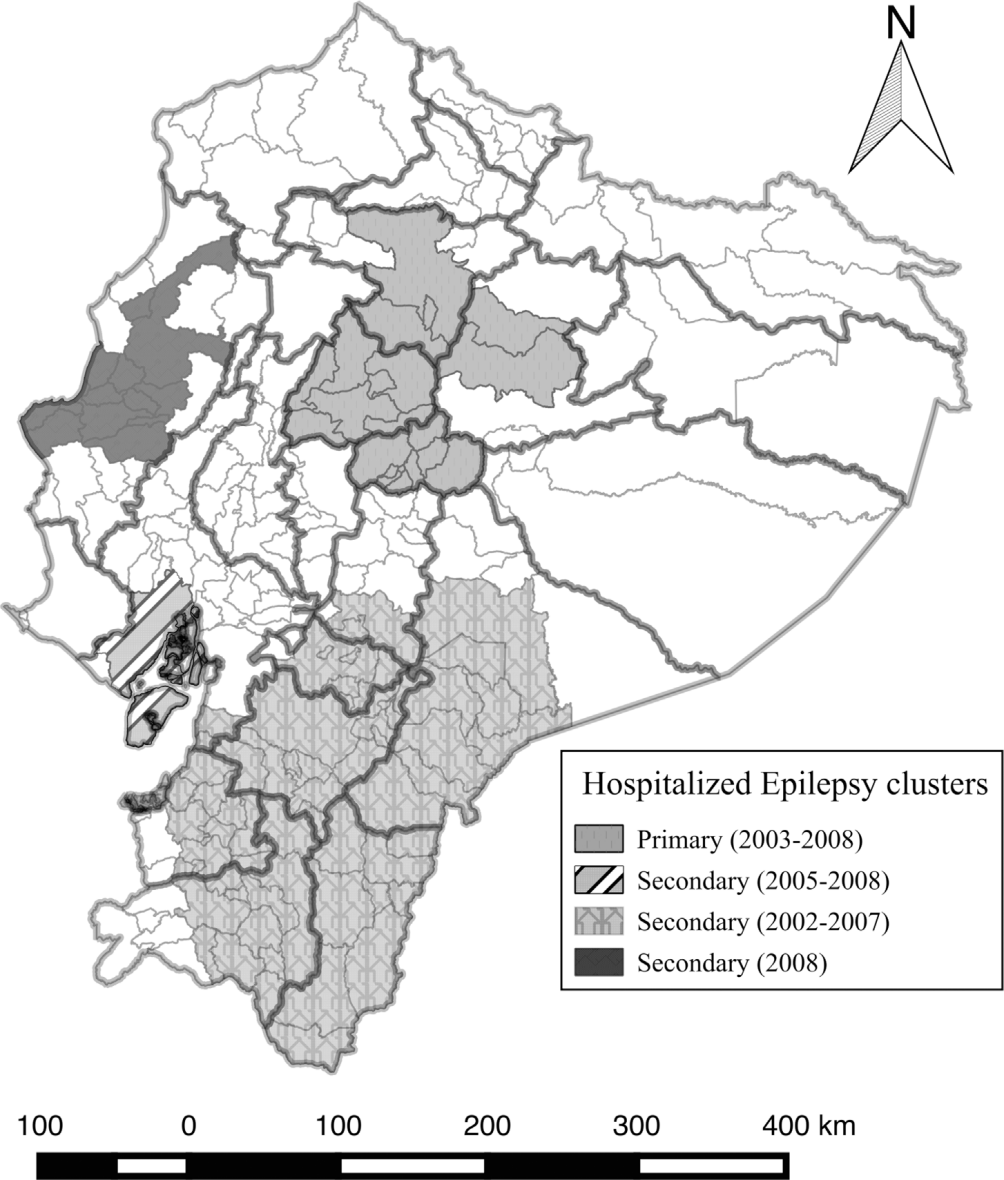
Significant space-time clusters of hospitalized epilepsy cases with up to a maximum of 25% of the total centroids included in the scanning window between 1996 and 2008.

Four clusters were detected for the iRR of epilepsy when up to a maximum of 25% of centroids were included in the scanning window (Fig 5). The most likely cluster extended from the center to the northern part of the country, involving municipalities from Tungurahua, Cotopaxi, Napo and Pichincha provinces. This cluster lasted between 2003 and 2008 with an iRR of 1.57 in contrast to centroids outside the cluster (p = 0.001). A secondary cluster was located in the Guayaquil municipality in Guayas province in the Southern coast of the country with an iRR of 1.69 (p = 0.001). This cluster existed between 2005 and 2008 with 1745 hospitalized cases when only 1070 cases were expected. A third secondary cluster was located in the southern part of the country in municipalities in the provinces of El Oro, Loja Zamora, Cañar, Azuay, Morona Santiago, and Chimborazo. This cluster was the biggest cluster found, and the iRR was 1.60 (p = 0.001) compared to the municipalities outside the cluster. This cluster existed from 2002 to 2007. Finally, in the western coast of the country, a secondary cluster covering 11 municipalities of Manabí province was detected in 2008, which had an iRR of 1.62 (p = 0.001).

**Fig 6:**
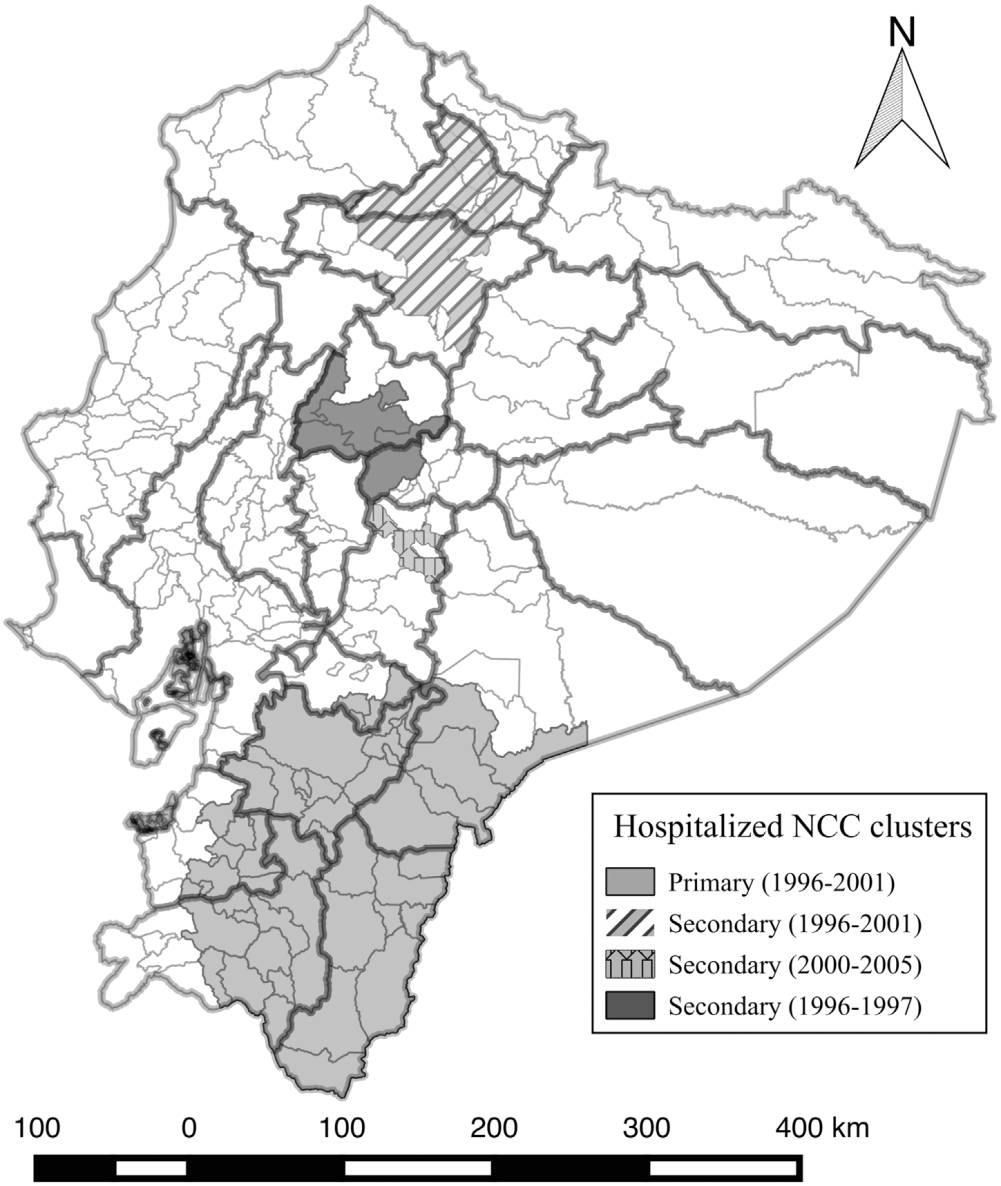
Significant space-time clusters of hospitalized NCC cases with up to a maximum of 25% of the total centroids included in the scanning window between 1996 and 2008.

Like for epilepsy, four significant clusters were identified for NCC. A main cluster was found in the southern part of the country, which existed from 1996 to 2001 with an iRR of 3.78 (p = 0.001) (Fig 6). Municipalities of El Oro, Loja, Zamora, Azuay and Morona Santiago provinces were part of this cluster within the given time frame. Likewise, in the central-northern part of the country, some municipalities from Imbabura, and Pichincha provinces were part of an important secondary cluster that lasted from 1996 to 2001. The iRR for the municipalities within this cluster was 2.75 (p = 0.001). Additionally, there was a secondary cluster in the municipality of Riobamba in Chimborazo Province in the middle of the country that lasted between 2000 and 2005. The iRR for this cluster was 3.15 (p = 0.001). Finally, besides the last zone, some municipalities from Cotopaxi and Tungurahua provinces were part of an additional cluster that had an iRR of 2.09 (p = 0.001) and had a relatively high incidence starting in 1996 until 1997.

### Potential indicators associated with the incidence of hospitalized epilepsy and neurocysticercosis cases

The analysis of several socio-economic and demographic variables affecting the IHC of epilepsy between 1996 and 2008 is presented in Tables 3 and 4. Table 3 presents the set of covariates selected by stepwise procedure and Table 4 presents the total set of variables included in this set of potential indicators and their partial contribution according to the BMA methodology. Posterior effect probabilities (*P*(*β≠* 0|*D*)) were included to show the evidence of an effect for each covariate when model uncertainty was incorporated. According to the results, the variables that significantly increased the IHC of epilepsy in municipalities were: the number of physicians per 10,000 inhabitants (PHYS) iRR = 1.045 (IC_95%_: 1.031–1.059), and the percentage of families having piped water (TUBWAT, not necessarily drinking water) iRR = 1.022 (IC_95%_: 1.014–1.029). On the other hand, the only variable that apparently led to a significant reduction in the IHC of epilepsy in municipalities was the percentage of houses having any kind of system to eliminate excrements (EXCR) iRR = 0.986 (IC_95%_: 0.979–0.993). The climatic variable (Zone) showed that municipalities located in the highlands presented an increased risk of epilepsy in contrast to municipalities located in tropical zones (iRR = 1.02 (IC_95%_: 0.855–1.207)) even though it was not statistically significant at a 5% level. For the BMA variable selection, out of 15 variables evaluated, 3 had substantial evidence (*P*(*β≠* 0|*D*)>90%) of being different from zero (Table 5); PHYS, TUBWAT, and EXCR. Overall, the BMA and stepwise selection methods yielded similar results.

**Table 3:** Indicator factors associated with the incidence of hospitalized epilepsy cases in Ecuador from 1996 to 2008 (negative binomial model chosen by AIC criterion).

Coefficients:	Estimate	Std. Error	z value	P-value
(Intercept)	-6.77	0.199	-33.95	<0.001
Zone (Tropical)	-0.16	0.088	-1.82	0.069
PHYS	0.04	0.007	6.37	<0.001
TUBWAT	0.02	0.004	5.53	<0.001
EXCR	-0.01	0.004	-3.85	<0.001
Dispersion parameter for Negative Binomial (3.50)				
AIC: 1769.8				

Legend: PHYS: Number of physicians per 10,000 inhabitants; TUBWAT: Percentage of dwellings with piped water; EXCR: Percentage of dwellings with some kind of system for eliminating excrements; AIC: Akaike Information Criterion; Std. Error: Standard Error.

**Table 4:** Indicator factors associated with the incidence of hospitalized epilepsy cases in Ecuador from 1996 to 2008 based on Bayesian model averaging (BMA).

Coefficients:	*P*(*β≠* 0|*D*)	Mean	SD
Intercept	100	-6.84	0.699
PHYS	100	0.05	0.008
TUBWAT	96.7	0.02	0.006
EXCR	94.0	-0.01	0.005

Legend: TUBWAT: Percentage of dwellings with piped water; PHYS: Number of physicians per 10,000 inhabitants; EXCR: Percentage of dwellings with some kind of system for eliminating excrements; SD: Standard deviation; P(*β≠*0|*D*): Posterior effects probability.

**Table 5:** Factors involved in the incidence of hospitalized neurocysticercosis cases in Ecuador from 1996 to 2008 based on a zero-inflated negative binomial regression model.

Count model	Coef.	Std. Err.	Z	P>|z|	[95% Conf. Interval]	
Intercept	-4.32	1.29	-3.35	0.001	-6.843	-1.790
ZONE (Tropical)	-1.72	0.170	-9.69	<0.001	-2.070	-1.37
SCHOOL	0.19	0.092	2.09	0.037	0.012	0.372
EXCR	-0.05	0.008	-6.62	<0.001	-0.068	-0.037
TUBWAT	0.02	0.008	1.98	0.047	0.0002	0.030
TECHASSIS	0.05	0.015	3.46	0.001	0.0222	0.080
%GLS	0.01	0.004	2.52	0.012	0.0024	0.019
EXTPOOV	-0.02	0.008	-2.33	0.020	-0.0362	-0.003
%RURAL	-0.01	0.005	-2.03	0.042	-0.0192	-0.0001
**Zero inflation**						
Intercept	-1.97	3.099	-0.64	0.525	-8.0451	4.102
EXCR	-0.06	0.028	-2.09	0.036	-0.1134	-0.003
PIG	-0.001	0.0003	-2.46	0.014	-0.0011	-0.0001
%RURAL	0.07	0.032	2.13	0.033	0.0056	0.131
log(alpha)	-0.70	0.1453	-4.74	<0.001	-0.9734	-0.403
Alpha	0.50	0.0730			0.3777	0.667

Legend: SCHOOL: Average number of years on formal education in people > = 24 years old; EXCR: Percentage of dwellings with some kind of system for eliminating excrements; TUBWAT: Percentage of dwellings with piped water; TECHASSIS: Percentage of land with technical assistance; %GLS Percentage of agriculture land dedicated to pastures; EXTPOOV: Percentage of extreme poverty; %RURAL: Percentage of rural population; PIG: Number of pigs (Criollo breed); Coef: coefficient; Std. Err.: Standard Error.

Table 5 presents the results of the potential indicator factors associated with NCC in hospitals. In the binary part, three covariates were chosen (EXCR, PIG, %RURAL). According to this selection procedure, the covariates that positively influenced the odds of hospitalized NCC cases in the communities were the implementation of systems for eliminating excrements (EXCR) (OR = 0.94; IC_95%:_ 0.89–1.0), and pig population (PIG) (OR = 0.999; IC_95%_: 0.999–1.0). In contrast, the higher the proportion of rural population in a community the lower the odds ratio of reporting NCC hospitalized cases (OR = 1.073 (IC_95%_: 1.01–1.14)).

On the other hand, for the count model, the variables that positively influenced the number of hospitalized cases were SCHOOL, TUBWAT, TECHASSIS, and %GLS, and the variables that were associated with a decrease in the number of hospitalized cases were Zone (Tropical), EXCR, EXTPOOV, and %RURAL. The temperate zone (highlands) had by far higher NCC cases compared to the tropical zones, so that the possibility of having a NCC diagnosis in Andean zone hospitals is higher (iRR = 5.6 times (IC_95%_: 3.95–7.92)). Variables such as, the schooling in municipalities (SCHOOL) (iRR = 1.212; IC_95%_: 1.012–1.451), the percentage of dwellings having piped water (TUBWAT), (iRR = 1.020;IC_95%_: 1.001–1.031), the proportion of farms with technical assistance (TECHASSIST) (iRR = 1.051; IC_95%_: 1.022–1.084), and the percentage of land dedicated to pastures (%GLS) (iRR = 1.010; IC_95%_: 1.002–1.019) were positively associated with an increase in the IHC of NCC. In contrast, covariates that negatively affected the number of NCC hospitalized cases in hospitals were: the percentage of dwellings having any system for eliminating excrements (EXCR) (iRR = 0.951; 0.934–0.964), extreme poverty (EXTPOOV) (iRR = 0.980; IC_95%:_ 0.965–0.997); and %RURAL population (iRR = 0.990; IC_95%:_ 0.9812–0.999). The implementation of systems for eliminating excrements (EXCR) reduced the IHC of NCC in average by 4.87% (IC_95%_: 3.6%–6.6%).

The model chosen for the iRR of epilepsy fitted better than the model without over-dispersion. The likelihood-ratio test for the over-dispersion parameter was significantly different from zero (p<0.001), meaning that Negative binomial regression was preferred over Poisson regression. For the case of NCC, when the zero-inflated negative binomial model was contrasted against the zero-inflated Poisson model, the likelihood-ratio test was significant in favour of taking into account the overdispersion (different from zero) (p<0.0001). In the same way, Vuong’s test confirmed that the assumption of zero inflation in the model for NCC was preferred over a model without this assumption (p = 0.02).

## Discussion

During the study period, NCC still had an impact on the general health status of the population in Ecuador. Our findings indicate that 6294 cases of NCC and 19821 cases of epilepsy were hospitalized between 1996 and 2008. Additionally, there was a significant increasing time-trend for IHC of epilepsy, but a decreasing time-trend for IHC of NCC overall. In contrast, within municipalities a positive linear relationship between both disorders was found. Also, the number of hospitalized cases (both for epilepsy and NCC) was related to some potential indicators evaluated. A general reduction in the IHC of both conditions was observed with an increasing percentage of systems to eliminate excrements. Moreover, the presence of pig production was related to the IHC of NCC.

### Epilepsy

In the case of epilepsy, according to the spatio-temporal analysis, all significant clusters of municipalities with high incidence of epilepsy existed between 2002 and 2008. The incidence risk ratio (iRR) of the epilepsy clusters were not so high (they ranged from 1.57 to 1.69), meaning that epilepsy can be almost classified as an endemic disorder in Ecuador. A possible explanation for the epilepsy clusters since 2002 is that during these years, health facilities could have improved in municipalities leading to a better coverage of hospitalization.

Clustered epilepsy municipalities were located in the Sierra region, mainly in the southern and some in the central-northern part of the country. Some of those municipalities were known as endemic for epilepsy and as epilepsy-NCC related zones, with some new clusters in the Sierra region that have not been pointed out before as endemic areas in previous studies [21;23;24;45]. The appearance of this new epilepsy cluster could also be related to the presence of other infectious and non-infectious diseases present in coastal tropical zones [46], but the evaluation of the newer clusters become urgent as this could be a strong predictor for NCC [4].

Based on two selection methods, there was a positive association between number of physicians and number of hospitalized cases of epilepsy. This effect occurs mainly in provincial capital cities where more health services exist and consequently specialized physicians are available to people. The implementation of systems for eliminating excrements, such as latrines, septic tanks, or sewage systems, seemed to have an impact on the occurrence of epilepsy, which suggests that a part of hospitalized epilepsy cases might be due to NCC or other fecal-related causes of epilepsy [47]. In this study period, the majority of the epilepsy cases were classified as non-specific cases (ICD codes: G408 and G409), and they were between 81% and 92% every year. As in other places, the majority of epilepsy cases apparently do not have a recognized etiological origin well identified [16;17]. However, not all types of acquired epilepsy can be attributed to NCC [4;16;47]. Other causes related to poverty, such as poor nutrition, neurological sequels due to infectious agents, and head traumas related to occupational accidents and violence can also contribute to the number of hospitalized epileptic cases [47–50]. The other variable was the percentage of dwellings with piped water. Having piped water not necessarily refers to water that is drinkable. According to estimations, in Ecuador, 30% of the piped water in urban zones is not potable water. [51].

### NCC

In Ecuador, the protocol to declare a patient with NCC depends on the diagnostic capacity of the neurology department, which in many cases, only exists in the main cities [52]. Based on national hospital data, 35.5% of the NCC cases were reported by the public sector, 39.4% were reported by the private sector and 23% by the systems of social security insurance. Furthermore, the appearance of clinical signs of NCC in patients might occur several months or even years after infection, so that some etiological factors could not have been measured correctly at the beginning of this study. Likewise, it is worth to mention that only a part of the cases of human cysticercosis are symptomatic, and therefore the statistical relationships found in this study are valid only for the hospitalized cases reported. A wider spectrum might be found with the addition of asymptomatic cases and people suffering from chronic headaches who do not often consult physicians, which only can be found in field studies[4;53–56].

NCC clusters in Ecuador apparently appeared earlier compared to epilepsy clusters and the majority of them existed between 1996 and 2001. Only one of them was identified from 2000 to 2005 in Chimborazo province and in some surrounding municipalities in Bolívar Province. This area presents ideal conditions for the *T*. *solium* life cycle, as it is located in the highlands with a high percentage of rural population and lack of basic services. In this cluster, more than 90% of the pig producers are smallholders [14]. According to the last Agricultural National Census carried out in 2000, 80.5% of the smallholders (≤10 pigs) are located in the Sierra region and 18.6% in the Coastal region. Porcine Cysticercosis has not been reported in slaughterhouses by the National Veterinary Services (Agrocalidad) since 2001 [57]. However, only 30% of slaughtered pigs in these facilities is provided by smallholder. [58].

Based on a zero-inflated negative binomial model, the percentage of rural population in municipalities was associated with a reduction in the IHC of NCC; so that urban zones increased their incidence in contrast with previous studies published [59]. Nowadays, the rural population in Ecuador accounts for less than 38% of the total population, which is a significant reduction compared to previous decades when the rural population was over 50%. Translocated rural communities tend to settle in slum zones of big cities [60]. However, rural and sometimes poor communities might have not been well represented in the data, as they may refrain from hospitalization due to the high costs of diagnosis and treatments [61]. This concern is a limitation of hospital-based registers [50]. In our study only 7% of patients appeared to belong to rural communities, although, if we consider the patients not living in the provincial capital cities this percentage increases to 26.7%. The structure of the data makes it difficult to differentiate people from peri-urban zones or slums, or semi-rural towns and communities from urbanized areas. On the other hand, housemaids and food vendors coming from endemic rural zones have a higher chance to be tapeworm carriers and can be at the origin of spreading *T*. *solium* infection among the urban population [18;62;63]. Another possible explanation is that traditional livestock systems are still preserved on a small scale in urban slums, although this presumption has not been quantified.

The presence of pigs was the most important positively associated with the appearance of hospitalized-symptomatic NCC cases. Industrialization of pig production, in many cases is not responsible for the increase in NCC cases. On the other hand, the presence of free roaming pigs has been associated with an increased risk for the occurrence of cysticercosis [24;64]. In Ecuador, despite 58.8% of pigs are raised in traditional production systems it has been estimated, that it only represents nearly 30% (50% in year 2000) of pork available in markets. [58].

In the negative binomial count model, eight variables were associated with the IHC of NCC. As in the case of epilepsy, the implementation of systems for eliminating excrements was involved in a reduction in the IHC of NCC. More in depth studies are needed to evaluate the real scale of those variables in the macro epidemiology of NCC, although some of them express the lack of quality in offering services.

### NCC & epilepsy

It has been mentioned that the difficulties of identifying the etiology of epilepsy could play an important role in the sub-notification of NCC [21;65;66]. The condition most commonly associated with NCC is epilepsy, but many cases of NCC are asymptomatic or manifest chronic headaches or other neurological disorders [4;54–56]. In *T*. *solium* endemic communities in Ecuador an important proportion of acquired epilepsy cases were due to NCC [21;45]. Although extrapolating this quantity to the current reality in the country may be biased; zones with an apparent increase of epilepsy cases may elucidate the origin of new suspected NCC cases [2;4]. Additionally, epilepsy and NCC in developing countries have been reported to be clustered [1;50;67], but the presence of imported cases has also been mentioned as an important factor in urban zones.

NCC underreporting might be due to a misdiagnosis in the epilepsy etiology. However, in our case both disorders were linearly related. This positive relationship is an indicator of an apparent constant relationship between epilepsy and NCC. This relationship has to be further studied and the meaning of this pattern has to be elucidated.

Additionally, given that the lack of sewage systems was demonstrated to be associated with an increase in the incidence of both conditions. Increasing the sewage systems could be used as an important control tool to reduce the incidence of the hospitalized cases. The installation of these systems, at municipal level varied from 20.6% to 96.3% of coverage with a median value of 68.5%, so there are still many municipalities that lack basic services.

The zone where the municipality was located was one of the principal indicators affecting the IHC of NCC and epilepsy. Municipalities located in temperate zones (highlands) had a significantly higher number of hospitalized NCC cases. From the BMA procedure, in the case of epilepsy, the posterior effect had a small probability (11.8%), so we argue that in tropical zones lack of appropriate diagnostic tools and specialized knowledge of health staff might make it difficult to properly identify NCC cases [48]. Likewise, the levels of coverage of basic services is lower in tropical zones of Ecuador [14]. Thus, the presence of the life cycle of *T*. *solium* in these zones traditionally considered to be NCC free cannot be ruled out.

A limitation of this study was that peri-urban zones where poverty belts of cities are frequently located could not be analyzed separately given that records do not use this residence category for patients. The conditions in peripheral zones can differ from city to city, thus the assumption that the origin of the hospitalized cases in big cities come from peripheral zones should not be extrapolated in all cases. As in other studies based on hospital data, rural communities might not be appropriately represented in the sample. This could be a major limitation of our study, and also because of the asymptomatic human cysticercosis cases, migraine-type and chronic headaches [4;54;56]. However, due to the fact that ambulatory cases do not offer reliable data, hospital data is a better attempt to represent the situation of the disease in a municipality. Another limitation ins this study might be the presence of duplicated cases in the data base. These cases might be due to the fact that a patient was hospitalized more than once, or because some NCC cases were diagnosed as epilepsy before. But given the mentioned limitations, the present results are still reliable due to their apparent representation of the municipalities in Ecuador, and because they are based on the appropriate statistical tools. So given the data constraints, the methods used to identify risk indicators and/or areas based on available data presents valuable results for veterinary and public health sectors at no cost. There is a need to re-evaluate the current situation for both disorders throughout the country as life conditions have been changing over time [26;30;68]. Given the recent changes in the organization of the public health sector, new trends need enough data collection-time to be evaluated again.

In conclusion, NCC might still have a relevant presence in Ecuador and might play an important role as a cause of acquired epilepsy in Ecuador [45]. Although the real burden of NCC is still unknown, we found that the hospitalization rate of patients with epilepsy has been increasing in recent years (Fig 4). Traditional NCC and epileptic endemic zones were recognized as high risk zones even though more recent clusters of both diseases seem to have appeared. Although the lack quality of basic services was related to the IHC in both disorders, one important finding of this study was that the implementation of systems for eliminating excrements helped to reduce the incidence of hospitalized cases of both epilepsy and NCC, which could be used as an indicator strategy for planning control programs. More specific studies linking human NCC with epilepsy and their respective factors in field conditions are needed to evaluate the prevalence of the disease in humans throughout the country and generate data that could be used for estimation of the burden disease.

## Supporting Information

S1 File STROBE Checklist. (DOC)

S2 File Access to electronic official databases. (DOCX)
