# Supplementary material for: Distribution and Potential Indicators of Hospitalized Cases of Neurocysticercosis and Epilepsy in Ecuador from 1996 to 2008
Source: PLoS Negl Trop Dis. 2015 Nov 18;9(11):e0004236. doi: 10.1371/journal.pntd.0004236 (PMC4651332; doi:10.1371/journal.pntd.0004236)
Supplement: S2 File — (DOCX) [file pntd.0004236.s002.docx]

Official database sources available at:

Morbidity of hospitalized reported cases per year of report:

<http://www.ecuadorencifras.gob.ec/estadisticas-de-camas-y-egresos-hospitalarios-bases-de-datos/>

Agricultural census of Ecuador:

<http://www.ecuadorencifras.gob.ec/censo-nacional-agropecuario/>

Social indicators (2001):

<http://indestadistica.sni.gob.ec/QvAJAXZfc/opendoc.htm?document=SNI.qvw&host=QVS@kukuri&anonymous=truehttp://indestadistica.sni.gob.ec/QvAJAXZfc/opendoc.htm?document=SNI.qvw&host=QVS@kukuri&anonymous=true&bookmark=Document/BM39>
